# Rate capability and electrolyte concentration: Tuning MnO_2_ supercapacitor electrodes through electrodeposition parameters

**DOI:** 10.1016/j.heliyon.2024.e41427

**Published:** 2024-12-21

**Authors:** Hamed Soltani, Hamed Bahiraei, Shahnaz Ghasemi, Mazdak Hashempour

**Affiliations:** a Department of Physics, Faculty of Science, Malayer University, Malayer, Iran; b Sharif Institute of Energy, Water and Environment, Sharif University of Technology, Azadi Avenue, P.O.Box11365-9465, Tehran, Iran

**Keywords:** Rate capability, Capacitance retention, Birnessite structure, Pseudocapacitance, Electrodeposition, δ-MnO_2_

## Abstract

Manganese dioxide (MnO_2_) is a well-known pseudocapacitive material that has been extensively studied and highly regarded, especially in supercapacitors, due to its remarkable surface redox behavior, leading to a high specific capacitance. However, its full potential is impeded by inherent characteristics such as its low electrical conductivity, dense morphology, and hindered ionic diffusion, resulting in limited rate capability in supercapacitors. Addressing this issue often requires complicated strategies and procedures, such as designing sophisticated composite architectures. This study introduces a straightforward and cost-effective approach to tune and enhance the rate capability of MnO_2_ pseudocapacitor electrodes fabricated via the electrodeposition method. Among the electrodeposition parameters, the deposition time and electrolyte concentration, which influence the mass loading, electrode thickness, microstructure, and electrochemical properties, were the primary focus. Various electrodes were prepared potentiostatically in a two-electrode cathodic electrodeposition setup on a Ni foam substrate in a KMnO_4_ aqueous electrolyte, with bath concentrations (in terms of Mn ion) of 0.01 and 0.1 M, and electrodeposition times ranging from 1 to 15 min. Optimal rate capabilities were achieved at low bath concentrations and deposition times, primarily due to the structural properties of electrodes prepared under such circumstances. While electrodeposition at a 0.1 M electrolyte concentration resulted in the formation of electrolytic MnO_2_ with high supercapacitive rate sensitivity, reducing the bath concentration to 0.01 M primarily led to the formation of birnessite δ-MnO_2_, capable of maintaining a reasonable specific capacitance in the range of approximately 90–100 Fg^–1^ with almost no sensitivity to the charging/discharging rate, as confirmed by galvanostatic charge-discharge (1–10 Ag^–1^) and cyclic voltammetry (10–100 mVs^−1^) examinations. Along with the positive structural impacts of the layered birnessite with large interlayer spacing, the porous morphology (vertically aligned two-dimensional interconnected columns) and low thickness (≈2 μm) of the electrode prepared at the lowest bath concentration and electrodeposition time (0.01 M in 1 min electrode) contributed to its fast ionic diffusion kinetics for pseudocapacitive charge storage and the consequent high rate capability.

## Abstract (author highlights)

• A simple and cost-effective method was used to improve the rate capability of MnO_2_ electrodes for supercapacitors.
• The electrodes were made by electrodeposition in KMnO_4_ electrolyte at concentrations of 0.01 and 0.1 M for 1–15 min".
• Electrode made at 0.01 M for 1 min, with a birnessite structure, showed fast ion diffusion and enhanced charge storage.
• These electrodes had a specific capacitance of 90–100 F g⁻^1^ and little change with different charge/discharge rates.".

## Graphical abstract

**Figure:**
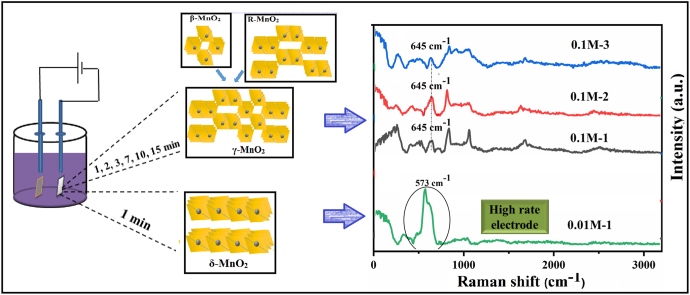

## 1 Introduction

Supercapacitors have garnered significant attention as energy storage devices owing to their unique properties, including high power density, rapid charge-discharge capabilities, and long cycle life [[1], [2], [3]]. At a fundamental level, supercapacitors can be categorized into two main types based on their energy storage mechanisms: electrical double-layer capacitors (EDLCs) and pseudocapacitors [[4], [5], [6]]. EDLCs, such as carbon materials, store energy electrostatically in the double-layer formed at the interface of the electrode material and the electrolyte [[7], [8], [9]], while pseudocapacitors, such as transition-metal oxides and conducting polymers, store energy through reversible superficial redox reactions of the electrode materials [10,11]. Compared to EDLCs, pseudocapacitors offer higher energy density due to their involvement in redox reactions [4,6,12].

Transition metal oxides and hydroxides are an important class of pseudocapacitive materials that offer promising properties such as high specific capacitance and reversible redox reactions [[13], [14], [15], [16], [17], [18]]. After the pioneering works of Trasatti [19] and Conway [20,21] on the pseudocapacitive behavior of RuO_2_, other metal oxides and hydroxides received immediate attention for this purpose, and MnO_2_ was among the earliest studied cases [[22], [23], [24], [25]]. MnO_2_ is particularly important in this context due to its high specific capacitance, reasonable reversibility of the surface redox reactions, and non-toxicity [[26], [27], [28]]. The electrochemical properties of MnO_2_ depend on various factors such as the crystal structure and defects, chemical composition, morphology, conductivity, porosity, and texture [[29], [30], [31], [32]]. Conventionally prepared MnO_2_ electrodes often exhibit limitations such as a low specific surface area, which can restrict the number of active sites available for redox reactions, as well as limited rate capability attributed to slow diffusion kinetics and restricted accessibility of electrolyte ions to these active sites [31,[33], [34], [35]]. Different strategies have been employed to enhance the power performance and rate capability of MnO_2_-based supercapacitors, addressing their inherent issues such as low specific surface area, slow ion diffusion kinetics, and limited access to active sites. These strategies include hybridization with carbon nanostructures, conducting polymers, and other transition metal oxides [[36], [37], [38]], as well as improving the morphology by varying synthesis conditions [[39], [40], [41]]. Furthermore, strategies that exploit specific crystallographic properties of MnO_2_ have been investigated. These approaches include utilizing wide channel structures or modifying the crystal phases to achieve desired planar orientation, interplanar spacing, and lattice porosity [[42], [43], [44]]. The overarching goal of all these approaches is to improve the overall power density, rate capability, and cycling stability of MnO_2_-based supercapacitors.

Among the aforementioned strategies to enhance the rate capability of MnO_2_-based supercapacitors, those relying on crystalline phase modification are of utmost importance. Unlike strategies involving composites and hybrids, which often require additional constituent materials, crystalline phase modification can directly impact both the electronic and ionic transport properties of the pseudocapacitive oxide, leading to significant and multifaceted performance enhancements. From a crystallographic point of view, manganese dioxide exists in various forms, known as typical MnO_2_ polymorphs, including, but not limited to, pyrolusite (β-MnO_2_), ramsdellite (R-MnO_2_), nsutite (γ-MnO_2_, an intergrowth of β- and R-MnO_2_), electrolytic manganese dioxide (EMD, ε-MnO_2_, an intergrowth of β- and R-MnO_2_), spinel (λ-MnO_2_), birnessite (δ-MnO_2_), and hollandite or cryptomelane (α-MnO_2_) [[45], [46], [47], [48]]. These phases exhibit different electrical conductivity, tunnel size, ion diffusion properties, and electrochemical stability, among other characteristics, and therefore, any change in the crystalline phase can significantly impact the electrochemical performance of MnO_2_ electrodes [[42], [43], [44]]. For example, β-MnO_2_ has a narrow 1 × 1 tunnel with a size range of 1.89–2.3 Å [42,[49], [50], [51]], which can hardly accommodate hydrated cations and thus usually does not exhibit promising capacitive behavior. In contrast, δ-MnO_2_ with a layered structure and interlayer spacing of greater than or equal to 7 Å [42,43,46,48,[52], [53], [54]], can easily accommodate electrolyte cations and thus exhibits fair notable capacitive behavior. Ghodbane et al. investigated the charge-storage mechanism in MnO_2_-based supercapacitors with various microstructures. MnO_2_ powders were synthesized using different methods, resulting in structures with 1D channels, 2D layers, or 3D tunnels. They found that the specific capacitance increased with larger cavity size and connectivity. The capacitive performance quality was reported to be in the following order: pyrolusite < Ni-todorokite < ramsdellite < cryptomelane < OMS-5 (manganese octahedral molecular sieve) < birnessite < spinel. The spinel form exhibited the highest capacitance, followed by birnessite. However, Ni-todorokite showed poor performance due to the presence of solvated Ni ions hindering ion diffusion. The study emphasizes that the electrochemical performance is mainly faradaic and correlated with ionic conductivity rather than specific surface area. Although the rate capability was not particularly assessed in this work, the emphasis posed on the diffusion kinetics in the lattice and the intermediate CV scan rate used in the measurements, suggest that α-MnO_2_, OMS-5, and δ-MnO_2_, should have superior rate capability [43]. This argument is supported by the work of Brousse et al. who compared the rate performance of several MnO_2_ polymorphs, including β-, γ-, λ-, and δ-MnO_2_, with poor rate capability reported for β-, γ- and λ- MnO_2_, and relatively better rate tolerance for δ-MnO_2_ [55], or the work of Cui et al., comparing γ-, δ- and α-MnO_2_, with superior rate performance of the δ- and α-MnO_2_ polymorphs and their mixture [56]. Devaraj and Munichandraiah investigated the influence of MnO_2_ crystallographic structures on electrochemical capacitance properties. Various crystal phases of MnO_2_ were synthesized in nano dimensions and evaluated for specific capacitance. α-MnO_2_ exhibited the highest specific capacitance (297 Fg^–1^), attributed to its wide tunnel size and large surface area, while β-MnO_2_ showed the lowest specific capacitance (9 Fg^–1^) due to its narrow tunnel size. Layered δ-MnO_2_ and α-MnO_2_ demonstrated the best capacitive behavior regarding both the specific capacitance and the rate capability, due to their large interlayer separation and tunnel size, respectively. In particular, the capacitance retention by increasing the specific current from 0.5 to 10 mA cm^−2^, was ≈85 % for α- and ≈75 % for the δ-MnO_2_ [42]. Sun et al. established a correlation between the specific capacitance of MnO_2_ and the percentage of effective Mn centers acting as active sites in Faradaic charge storage. This relationship is based on the correlation between tunnel structure–crystallization behavior correlation, distinguishing between adsorption/desorption and insertion/extraction processes. Different MnO_2_ crystallographic forms exhibit varying specific capacitance values due to differences in effective Mn center utilization in size-limited tunnels. Decreasing crystal size increases the percentage of effective Mn centers, leading to higher specific capacitance values in MnO_2_-based electrode materials [44]. Zhao et al. investigated the electrochemical performance of α-, β- and δ-MnO_2_, prepared under various hydrothermal synthesis conditions. Their findings indicated that the specific capacitances of the MnO_2_ samples are ordered as follows: δ- > α- > α + β > β-MnO_2_. The rate sensitivity assessment also revealed that α-MnO_2_ is the most rate capable electrode [57].

The realization of an appropriate rate capability, in addition to a rational strategy, requires a viable and effective implementation method. In practice, deploying these strategies translates into the synthesis methods of the active material. The synthesis of MnO_2_ nanostructures encompasses various techniques, including hydrothermal [[58], [59], [60], [61]], chemical vapor deposition (CVD) [62], vapor–liquid–solid (VLS) [49], sol-gel synthesis [63,64], pulsed laser deposition [65], template-assisted synthesis [66,67], and electrodeposition [[68], [69], [70], [71], [72], [73], [74]]. The electrodeposition method offers significant advantages over other methods, For example hydrothermal synthesis which is effective method for producing well-defined crystalline nanostructures, requires high temperature and pressure conditions, that makes it energy-intensive, time-consuming, and limited in scalability. Also chemical vapor deposition (CVD), produces high-purity, uniform thin films with excellent substrate adhesion but requires expensive precursors, vacuum systems, and high-temperature processing, which increases costs and limits versatility. Among these, electrodeposition is notable for its techno-economic advantages, simplicity, cost-effectiveness, rapid deposition at ambient conditions, precise control over parameters, and a binder- and template-free approach [69,[75], [76], [77]]. However, its extensive range of operating parameters, such as deposition technique and power supply regime (static or dynamic, galvano or potentio, continuous or pulsed, DC or AC, etc.), potential level and overpotential, current density, bath composition and concentration, pH, additives and complexing agents, temperature, agitation level, deposition duration, presents challenges alongside its versatility [[78], [79], [80]]. Literature offers insights into the assessment of electrodeposition operating parameters, including the deposition technique and mode [75,[81], [82], [83], [84], [85]], duration [86,87], voltage [73,88], current density [40,87,89,90], temperature [89,91], pH [89], electrolyte type and bath composition [40,89,[92], [93], [94], [95]], additives [77,[96], [97], [98]], and their influence on the capacitive behavior of MnO_2_ coatings. However, there is a scarcity of reports regarding the critical role of the electrodeposition bath concentration in this regard [40,89,92]. Ye et al. investigated the electrodeposition of nanostructured MnO_2_ on carbon fiber paper (CFP) to tailor its morphology for enhanced supercapacitor performance. By adjusting H_2_SO_4_ concentration and current density, they obtained four distinct morphologies: nanospheres, nanosheets, nanoflowers, and nanorods, all possessing a γ-MnO_2_ structure with different proportions of pyrolusite and ramsdellite. They achieved a high specific capacitance of 362 Fg^−1^ at 0.5 Ag^−1^ and 44 % capacitance retention at 5 Ag^−1^ [40]. Wei et al. explored the morphology evolution of MnO_2_ nanostructures through anodic electrodeposition from acetate-containing aqueous solutions. By adjusting deposition parameters, particularly the bath concentration, various coating morphologies including, petal- and flower-like structures, clusters, and interconnected nanosheets were obtained, all possessing an antifluorite-type structure. The study revealed that the rich morphology of MnO_2_ is influenced by the supersaturation ratio of the electrolyte components at the electrode/electrolyte interface, impacting reaction kinetics. Electrochemical analysis highlighted the superior performance of oriented nanostructures, such as columnar and interconnected nanosheet architectures, attributed to their enhanced manganese oxide utilization [89]. Babakhani and Ivey investigated the effects of electrodeposition conditions on the electrochemical behavior of manganese oxide electrodes. Anodic deposition from acetate-containing aqueous solutions yielded various morphologies such as continuous coatings, rod-like structures, aggregated rods, and thin sheets, all with an antifluorite-type crystal structure. Morphology control was achieved by adjusting the supersaturation ratio, impacting nucleation and growth processes. Electrochemical analysis revealed that electrodes with rod-like and thin sheet morphologies exhibited superior performance, with thin sheets demonstrating the highest specific capacitance (≈230 Fg^−1^) and capacitance retention (≈88 % after 250 cycles) [92].

The present study aims to provide a simple yet effective solution to address the low rate capability of MnO_2_ pseudocapacitor electrodes. While significant progress has been made over the past decades in enhancing the specific capacitance of MnO_2_ electrodes, there remains a noticeable gap in addressing their rate tolerance. Our focus is on this less-studied aspect, which is critical for supercapacitors –devices primarily designed for power applications. The proposed approach involves a novel combination of a synthesis method –electrodeposition– and a key synthesis parameter –electrodeposition bath concentration– along with structural modifications, a combination infrequently explored in previous research. Using a Ni foam substrate and a KMnO_4_ aqueous electrolyte for electrodeposition, we found that variations in bath concentration and deposition time significantly influenced factors such as mass loading, electrode thickness, crystal structure, and morphology, with clear impacts on rate capability. For an in-depth understanding of the potential modifications of the electrodes by electrodeposition parameters, a wide range of microstructural (X-ray diffraction (XRD), Raman spectroscopy, field-emission scanning electron microscopy (FE-SEM)) and electrochemical (cyclic voltammetry (CV), galvanostatic charge-discharge (GCD), electrochemical impedance spectroscopy (EIS) characterization techniques were employed. Lower bath concentrations and shorter deposition times optimized rate capabilities. Our findings indicate that, in contrast to previous reports, variations in electrolyte concentration can alter the crystal structure of the electrodeposited MnO_2_ film. While 0.1 M electrolyte concentration produced electrolytic ε-MnO_2_, reducing it to 0.01 M led to δ-MnO_2_ formation, exhibiting superior rate performance due to its layered birnessite structure, large interlayer spacing, porous morphology, and low thickness. The best MnO_2_ electrodes in this respect demonstrated a rate capability of more than 90 % when increasing the rate by an order of magnitude, whether using CV or GCD for characterization. This study highlights the potential of simple yet strategic manipulation of electrodeposition parameters, particularly electrolyte concentration, to significantly improve the rate capability of MnO_2_ supercapacitor electrodes, thereby opening new avenues for enhancing the performance of supercapacitors in practical applications.

## 2 Experimental

### 2.1 Preparation of MnO_2_ electrodes

**Fig. 1:**
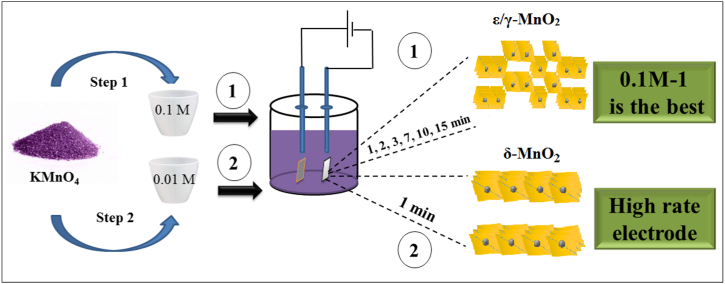
Schematic representation of the MnO_2_ electrode preparation procedure for different concentrations and different electrodeposition times.

The electrodeposition setup used for electrode preparation was a two-electrode cell in a potentiostatic regime. The working electrodes were 1 × 1 cm Ni foams with a thickness of 1.5 mm, ≈95 % porosity, ≈800 m^2^ m^−3^ volumetric surface area, and a mass of approximately 70 mg. The selection of Ni foam as the substrate was driven by its suitability for electrodeposition due to good electrical conductivity, mechanical integrity, electrochemical stability and compatibility with the electrolytes of both the electrodeposition and capacitive characterization stages [99]. The counter electrode was a 1 × 1 cm Pt sheet. In a KMnO_4_ aqueous electrolyte, the MnO_2_ nanolayers were cathodically deposited under a constant potential of 10 V on the Ni foam, which was ultrasonically cleaned with acetone, rinsed with distilled water and dried at 60 °C for 1 h. Analytical-grade chemicals (Merck) and distilled water were utilized for electrolyte preparation, the pH of the electrolyte was not modified, and the electrodeposition was conducted at room temperature without stirring. Following, electrodeposition, the coated electrodes underwent rinsing with distilled water and drying at 60 °C for 1 h. Electrodeposition variables included (a) electrolyte concentration: 0.1 M and 0.01 M, and (b) deposition time: 1, 2, 3, 7, 10, and 15 min. The resulting electrodes were labeled as y-M-x, where y represented the electrolyte concentration (0.1 and 0.01) and x represented the deposition time (1, 2, 3, 7, 10, and 15). The loaded mass of MnO_2_ was calculated as the difference in mass of the Ni foam before and after electrodeposition, rinsing, and drying. Fig. 1 illustrates the schematic diagram of the electrode preparation process.

### 2.2 Materials characterizations

The surface morphology of electrodeposited MnO_2_ films was characterized using field emission scanning electron microscopy (FE-SEM, TeScan – Mira III) equipped with energy-dispersive X-ray spectroscopy (EDX). EDX was employed for the composition analysis of MnO_2_ films. The crystallographic structure of the coatings was assessed via X-ray diffraction (XRD, Malvern Panalytical, Cu Kα; λ = 0.15406 nm). Raman spectra of MnO_2_ thin films were recorded using a Teksan (Takram) Raman spectrometer with a 532 nm excitation laser light source and 1 mW excitation laser power.

### 2.3 Electrochemical measurements

The electrochemical capacitive behavior of the MnO_2_ electrodes was investigated through cyclic voltammetry (CV), galvanostatic charge-discharge (GCD), and electrochemical impedance spectroscopy (EIS) in a 1 M KOH electrolyte at room temperature. The experiments were conducted using an OrigaFlex-OGF500 Potentiostat/Galvanostat system equipped with a frequency response analyzer (FRA). A three-electrode cell configuration was employed, comprising MnO_2_ deposits on a Ni foam substrate as the working electrode, a Pt sheet as the counter electrode, and an Ag/AgCl electrode as the reference electrode. CV experiments covered a potential window of 0–550 mV_Ag/AgCl_ at scan rates of 10, 20, 50, 80 and 100 mVs^−1^. GCD experiments were performed within the potential range of 0–480 mV_Ag/AgCl_ with specific currents of 1, 2, 3, 5, and 10 Ag^–1^. EIS measurements were conducted at the open circuit potential (OCP) using an excitation signal with a 5 mV amplitude in the frequency range from 50 kHz to 50 mHz, sampling 10 points per decade.

The specific capacitance values, $C_{s}$ (Fg^−1^), were calculated using both CV and GCD results based on the following equations [[100], [101], [102], [103]]:

(1) $$C_{s}=\frac{\frac{1}{2}\oint IdV}{\nu \Delta V\;m}$$

(2) $$C_{s}=\frac{2\;I\int Vdt}{m\;{\Delta V}^{2}}$$

In equation (1) corresponding to CV experiments, I is the variable current (A) as a function of the potential V, ΔV is the nominal potential window of the CV (V), the term ∮IdV is the total area enclosed in the voltammogram of the single electrode in a complete forward-backward cycle, v is the scan rate (V s^−1^), and m is the active mass of the single electrode (g).

In equation (2) corresponding to GCD experiments, I is the constant discharge current (A), ΔV is the effective discharge potential window after the ohmic drop (V), the term ∫Vdt represents the area under the discharge curve after the ohmic drop, and m is the active mass of the single electrode (g). Furthermore, the specific energy (gravimetric energy density, $E_{s}$) and specific power (gravimetric power density, $P_{s}$) were evaluated based on the GCD results using equations (3), (4)) respectively [[100], [101], [102], [103]]:

(3) $$E_{s}=\frac{1}{2}C_{s}{\Delta V}^{2}$$

(4) $$P_{s}=\frac{E_{s}}{\Delta t}$$

where ΔV is the effective discharge potential window after the ohmic drop (V), and Δt is the time interval (s) corresponding to ΔV.

## 3 Results and discussion

### 3.1 Microstructural characterization

**Fig. 2:**
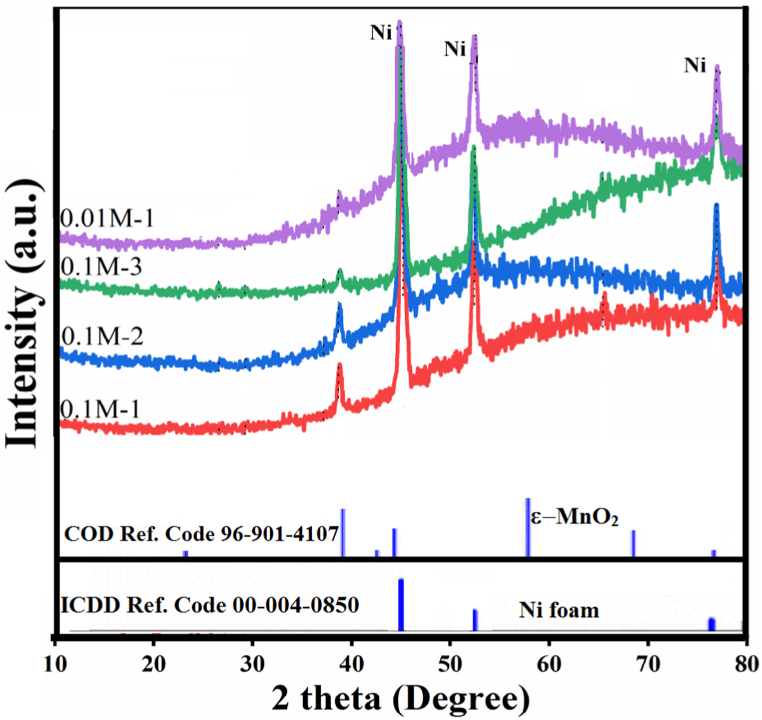
XRD patterns of 0.1-M-x (1, 2, and 3), and 0.01-M-1 electrodes.

Fig. 2 depicts the XRD patterns of various MnO_2_ electrodes electrodeposited under different electrolyte concentrations and deposition times (0.1-M-1, 0.1-M-2, 0.1-M-3, and 0.01-M-1). The three prominent peaks observed in all spectra, at 2θ ≈ 44.5°, 51.8°, and 76.4°, correspond to the diffractions of the Ni substrate, namely, (111), (200), and (220) planes, respectively, as per ICDD Ref. Code 00-004-0850 [104]. All 0.1-M-x electrodes exhibit a peak at 2θ ≈ 38°, with the peak intensity decreasing as a function of deposition time. Notably, the 0.1-M-1 electrode additionally displays a peak at 2θ ≈ 67°, a characteristic which is dramatically suppressed or completely absent in 0.1-M-2 and 0.1-M-3, following the observed relationship between peak intensity and deposition time for the 38° peak. These two peaks align more fittingly with the ε polymorph of MnO_2_ (38° and 67° peaks corresponding to (100) and (110) planes, respectively, as per COD Ref. Code 96-901-4107 [105]. As elucidated by Chabre and Pannetier [46], recent literature predominantly associates ε-MnO_2_ with electrochemically prepared MnO_2_. Traditionally, MnO_2_ phases significant for industrial battery applications –namely, γ-MnO_2_ and ε-MnO_2_– were collectively categorized under the nsutite family; however, in contemporary contexts, nsutite is more closely associated with γ-MnO_2_. Both γ-MnO_2_ and ε-MnO_2_ exhibit disordered intergrowth of Pyrolusite (β-MnO_2_) and Ramsdellite (R-MnO_2_) [46,48], albeit to different extents and with varying structural faults [106,107], and their XRD patterns are characterized by a few relatively low-intensity lines over a diffuse background, bearing similarities to both Pyrolusite and Ramsdellite [46]. Explaining the weak diffraction peaks observed in 0.1-M-x electrodes, in addition to the intrinsic characteristics of ε/γ-MnO_2_ phases mentioned above, one must consider the overall poor crystallinity of as-deposited films and their presumably small crystallite size [59,[108], [109], [110]]. The diminishing intensity of both the 38° and 67° peaks with deposition time implies that only the initially deposited layer in direct contact with the substrate, i.e., the nucleation stage layer, has been formed in a relatively crystalline manner, while the subsequent layers are more amorphous. Additionally, the top layers may mask the XRD signal from the underlying layers. It is postulated that the crystalline growth of MnO_2_ is kinetically slow [[111], [112], [113]]. Consequently, during prolonged electrodeposition periods at high manganese ion concentrations, the deposition rate, driven by the large electrochemical driving force, exceeds that needed for crystalline growth, leading to the formation of more amorphous layers. The absence of clear MnO_2_ diffraction peaks in the 0.01-M-1 electrode can be attributed to 1) the low amount of deposited material due to the short deposition time of 1 min and low electrolyte concentration, and 2) the formation of a different MnO_2_ polymorph compared to 0.1-M-x electrodes (i.e., ε/γ-MnO_2_ phases), exhibiting a more amorphous nature even at the nucleation stage.

**Fig. 3:**
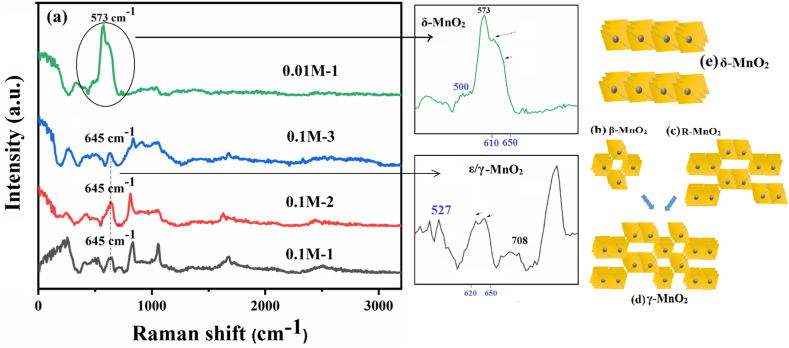
Raman spectra of 0.1-M-x (x = 1, 2, and 3) and 0.01-M-1 electrodes with insets magnifying the important regions (a), and the crystal structure of β-MnO_2_ (b), R-MnO_2_ (c), ε/γ-MnO_2_ (d) and δ-MnO_2_ (e).

To gain further structural insight into the differences between the 0.1-M-x and 0.01-M-x electrodes, Raman spectroscopy was conducted using a 532 nm excitation laser light source (Fig. 3a). Various vibration modes of Mn–O bonds in MnO_6_ octahedra, as well as combinational modes and collective vibrations of MnO_6_ octahedra in manganese oxides, are known to produce distinct Raman bands, particularly in the frequency range of 450–750 cm^−1^ [[114], [115], [116], [117], [118], [119]]. The 0.01-M-1 electrode exhibits several Raman bands in that region. Compared to the 0.1-M-x electrodes, the 0.01-M-1 electrode displays a strong peak at 573 cm^−1^, along with a pair of bands at 610–650 cm^−1^ and a lower-frequency a lower-frequency weak band at about 500 cm^−1^. These characteristics are indicative of birnessite, δ-MnO_2_, as the primary manganese oxide polymorph in this sample [117,[120], [121], [122], [123]]. The 0.1-M-x electrodes lack the band in the 570–580 cm^−1^ region and are primarily characterized by a doublet band in the 620–650 cm^−1^ region, along with some weak features at 527 and 708 cm^−1^. These characteristics suggest a combination of β-MnO_2_ and R-MnO_2_. β-MnO_2_ is characterized by two main bands at ≈540 cm^−1^ and ≈665 cm^−1^, while R-MnO_2_ is characterized by two main bands at ≈522 cm^−1^ and ≈575 cm^−1^, along with some weaker features at 630 cm^−1^ and 648 cm^−1^ [114,[116], [117], [118],^121^].

While MnO_6_ octahedra are the common building blocks of all the aforementioned MnO_2_ phases, variations in their spatial arrangement, packings, and interconnections (edge/corner sharing) result in a variety of polymorphs. β-MnO_2_ consists of single chains of edge-sharing MnO_6_ octahedra, connected to neighboring chains at the corners, forming a (1 × 1) tunneled structure (Fig. 3b). R-MnO_2_ is composed of double chains of edge-sharing MnO_6_ octahedra, with a specific interconnection that produces a (1 × 2) tunneled structure (Fig. 3c). ε/γ-MnO_2_ phases are intergrowths of β-MnO_2_ and R-MnO_2_, and their corresponding tunneled frameworks appear as a mixture as well (Fig. 3d), often wider than (1 × 1) and more resembling (1 × 2). δ-MnO_2_ consists of 2D planes of MnO_6_ octahedra separated by gaps between the layers, creating 2D tunnels (1 × ∞), as shown in Fig. 3e [42,[45], [46], [47], [48],^124^]. The tunnels in β-MnO_2_ are relatively small, hindering the accommodation and facile movement of solvated ions. Consequently, in electrochemical applications that rely on ionic diffusion, β-MnO_2_ is kinetically unfavored. In contrast, tunnels in R-MnO_2_ are twice as wide as those in β-MnO_2_, resulting in faster electrochemical performance [42,43,46,48]. It is important to note, however, that R-MnO_2_ is not commonly found as a pure phase and is often mixed with other phases, such as β-MnO_2_, forming the so-called electrolytic MnO_2_ (ε/γ phases) [46,125]. Such mixed structures benefit from the wider tunnels of R-MnO_2_ and are therefore favored for diffusional electrochemical applications. Finally, δ-MnO_2_, possessing 2D tunnels with an opening ≥7 Å, stands out as the fastest MnO_2_ framework for electrochemical applications involving ionic diffusion [42,43,46,48,[52], [53], [54]].

**Fig. 4:**
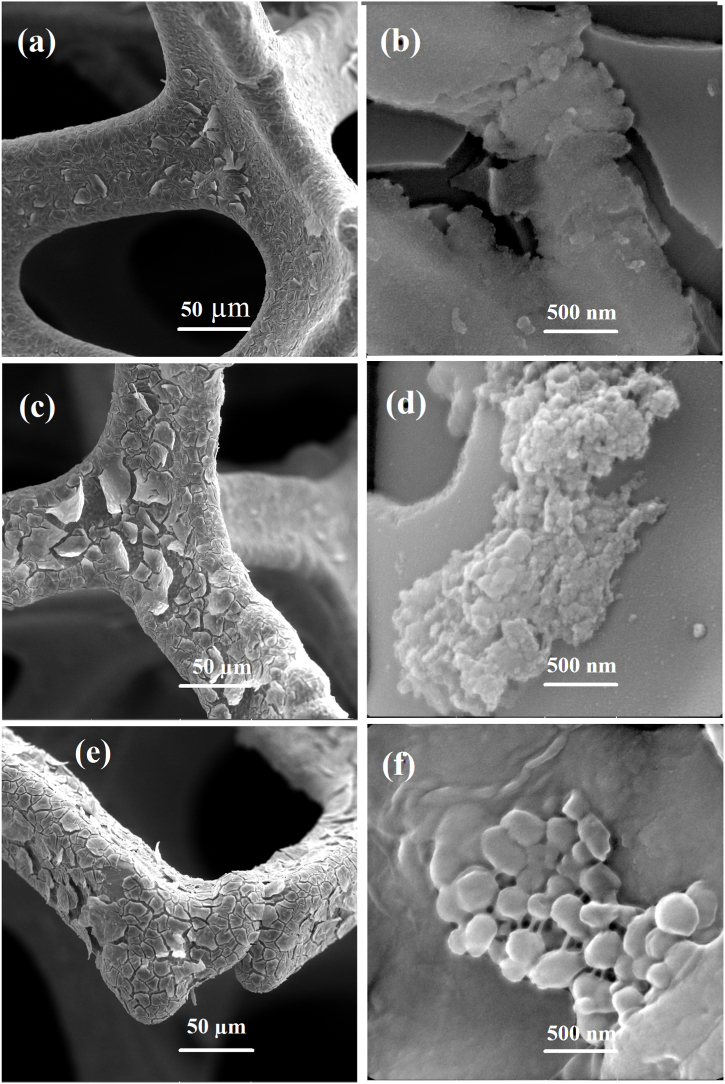
FE-SEM images of 0.1-M-1 electrode (a–b), 0.1-M-2 electrode (c–d), 0.1-M-3 electrode (e–f), at different magnifications.

**Fig. 5:**
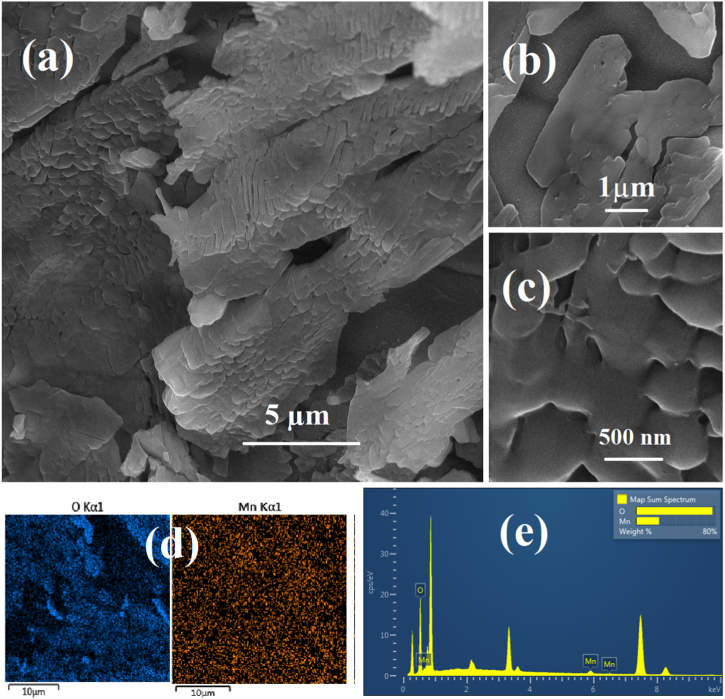
FE-SEM images of 0.01-M-1 at different magnifications (a–c), elemental mapping of oxygen and manganese in the 0.01-M-1 electrode (d), and EDX analysis of 0.01-M-1 electrode (e).

Fig. 4, Fig. 5 depict FE-SEM morphological studies of the 0.1-M-x and 0.01-M-x electrodes, respectively. The 0.1-M-x electrodes exhibit a compact coating that increases in thickness with longer deposition times. The thicknesses are approximately 1.5-, 2.5- and 3.5 μm for 1-, 2- and 3 min deposition times, respectively (Fig. 4a–c, e). Commonly observed across all 0.1-M-x electrodes are cracks, likely resulting from the dewatering process and subsequent shrinkage of the coatings during drying. Thicker layers, such as 0.1-M-2 and 0.1-M-3 (Fig. 4c–e), appear more susceptible to adhesion loss during the drying-shrinkage-cracking sequence. Higher magnification images (Fig. 4b–d, f) do not reveal distinct nanoscale characteristics for the coatings, suggesting a dense, monolithic, and flat structure. However, at extended deposition times, some fine aggregates of nanoparticles seem to form on top of the dense and flat coatings (Fig. 4d–f). The nanoparticles' morphological distinction from the electrodeposited layer may indicate a different formation mechanism. For instance, chemical precipitation induced by local pH variations near the working electrode (cathodic alkalinization) could be a contributing factor [[126], [127], [128], [129]]. Formation of a secondary nanoparticle morphology along the primary phase is also reported in other works, e.g., in the work of Jeong et al. [91], and has been attributed to increased nucleation sites.

The dilution of the electrodeposition bath appears to have significantly altered the coating morphology, as observed in the 0.01-M-1 electrode (Fig. 5a–c). The approximately 2 μm layer exhibits a non-monolithic structure composed of vertically grown 2D columns. Among various MnO_2_ polymorphs, a 2D layered nanostructure is primarily characteristic of δ-MnO_2_ [42,130,131]. However, at the microscale, various morphologies have been reported for δ-MnO_2_, including cloudlike, spongy, flowerlike, acicular, and disk-like structures, depending on the synthesis procedure, mechanism, chemical environment, and adjacent nucleation sites [43,50,[132], [133], [134], [135], [136], [137], [138], [139], [140], [141]]. The columnar growth of the layer suggests a preferred orientation and surface energy anisotropy of different facets within the ceramic crystal [142]. The columnar and non-monolithic structure creates interconnected 2D pores that extend through the depth and thickness of the layer. Based on our approximate calculation of the oxide film apparent volumetric density relying on the mass loading (discussed later in the last section) and thickness data, the 0.01-M-1 electrode (≈1.25 g cm^−3^) has about 1/4 of the theoretical density of pure solid MnO_2_ (5.026 g cm^−3^) and about 1/2 of the density of 0.1-M-1 electrode (≈2.5 g cm^−3^). This indirectly demonstrates the increased pore development in the MnO_2_ film due to decreased electrodeposited bath concentration. These pores provide pathways as wide as several tens of angstroms, facilitating the penetration of the electrolyte. Therefore, reducing the electrolyte concentration appears to lead to the formation of a porous structure along the nanolayers, enabling the access of electrolyte ions to sublayers of the active material. The composition of the 0.01-M-1 coating was assessed via EDX mapping and point analysis (Fig. 5d and e), confirming the purity of the layer, consisting solely of manganese and oxygen. Supporting literature corroborates these findings, emphasizing the relationship between electrolyte concentration, deposition kinetics, and resulting electrode morphology. For instance, insightful studies by the Ivey group [89,92,143] using different electrodeposition baths (manganese nitrate, acetate, etc.) demonstrate that higher Mn concentrations favor continuous, compact coatings. However, by decreasing the Mn concentration, the continuity of the coated films is gradually lost, and discrete oxide clusters become dominant. At extremely low Mn concentrations, these discrete clusters tend to form in morphologies with strong preferred orientation and remarkable porosity, such as submicron-scale columns. This aligns well with our observations, where the lower KMnO_4_ concentration resulted in a porous and layered birnessite, while higher concentrations produced denser structures.

### 3.2 Electrochemical characterization

**Fig. 6:**
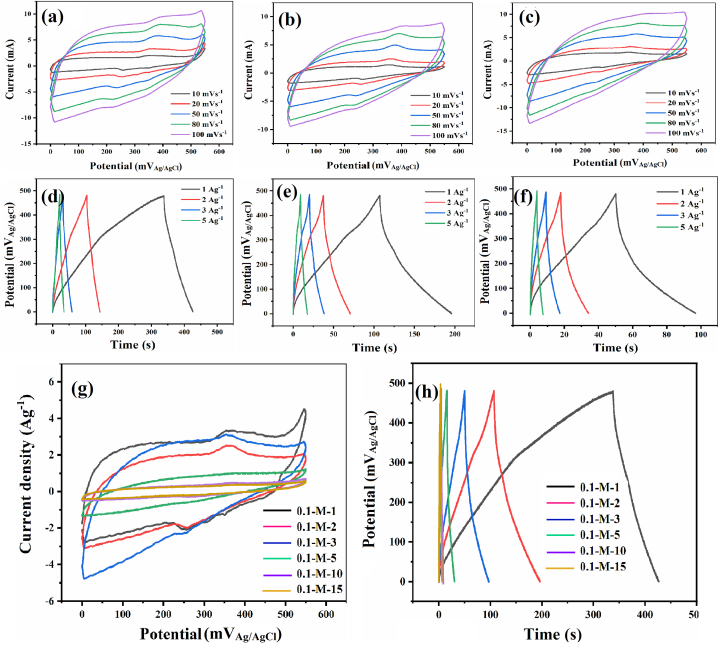
CV profiles of 0.1-M-1 (a), 0.1-M-2 (b), and 0.1-M-3 (c) electrodes at 10–100 mVs^−1^ scan rates. GCD profiles of 0.1-M-1 (d), 0.1-M-2 (e), and 0.1-M-3 (f) electrodes at 1–5 Ag^–1^ specific currents. CV comparison of 0.1-M-x (1, 2, 3, 7, 10 and 15) electrodes at 20 mVs^−1^ (g), GCD comparison of 0.1-M-x (1, 2, 3, 7, 10 and 15) electrodes at 1 Ag^–1^ (h).

**Fig. 7:**
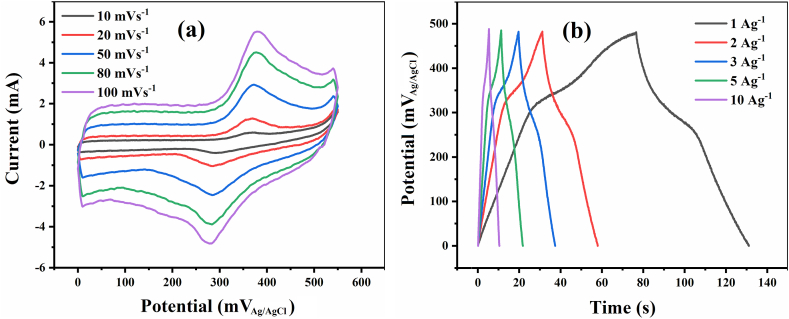
CV profiles of the 0.01-M-1 electrode at 10–100 mVs^−1^ scan rates (a), GCD profiles of the 0.01-M-1 at 1–10 Ag^–1^ specific currents (b).

Electrochemical characterization of the active materials and the influence of deposition time and electrolyte concentration on their capacitive performance were investigated using CV, GCD, and EIS techniques in a three-electrode setup, as illustrated in Fig. 6, Fig. 7, Fig. 9.

Fig. 6 presents the CV and GCD results of the 0.1-M-x electrodes. In Fig. 6a–c, the rate sensitivity of each electrode (for x = 1, 2, and 3) can be observed across a scan rate range of 10–100 mVs^−1^, while Fig. 6 g compares the voltammetric behavior of different electrodes (for x = 1, 2, 3, 7, 10, and 15) at a fixed scan rate of 20 mVs^−1^. For a quantitative comparison, the specific capacitance of all electrodes (x = 1, 2, 3, 7, 10, and 15) at various scan rates is presented in Table 1. Additionally, Table 1 includes the ratio of a low specific capacitance (at a high scan rate, i.e., 100 mVs^−1^) to a high one (at a lower scan rate, i.e., 10 mVs^−1^), serving as a measure of the electrode's capability to retain capacitance as the charging rate increases. A higher percentage ratio indicates a greater rate capability of the electrode.

**Table 1:** The specific capacitance and rate capability of the prepared electrodes based on the CV characterization.

Electrode	C (Fg^−1^) at 10 mVs^−1^	C (Fg^−1^) at 20 mVs^−1^	C (Fg^−1^) at 50 mVs^−1^	C (Fg^−1^) at 80 mVs^−1^	C (Fg^−1^) at 100 mVs^−1^	Rate capability (%)
0.1-M-1	213	214	157	142	140	66
0.1-M-2	140	126	99	88	83	59
0.1-M-3	80	65	48	41	39	49
0.1-M-7	–	27	–	–	–	–
0.1-M-10	–	12	–	–	–	–
0.1-M-15	–	10	–	–	–	–
0.01-M-1	82	91	89	90	89	105

**Fig. 8:**
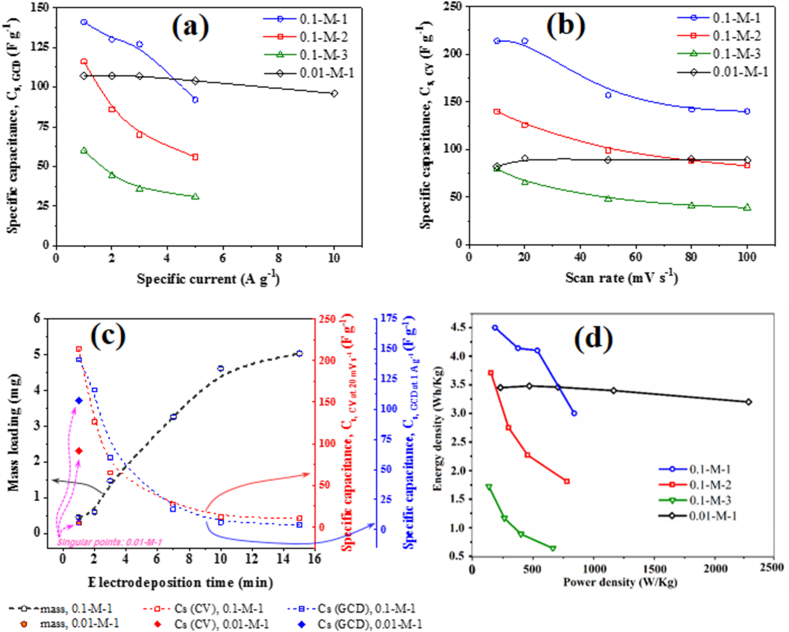
The specific capacitance of 0.1-M-x (1, 2, and 3) and 0.01-M-1 electrodes as a function of the specific current in GCD experiments (a), the specific capacitance of 0.1-M-x (1, 2, and 3) and 0.01-M-1 electrodes as a function of the scan rate in CV experiments (b), the mass loading and specific capacitance vs. electrodeposition time of MnO_2_ (c). the ragone plot of 0.1-M-x (1, 2, and 3) and 0.01-M-1 electrodes (d).

The 0.1-M-1 electrode exhibited the highest gravimetric capacitance, reaching approximately 214 Fg^−1^ at scan rates between 10 and 20 mVs^−1^. The gravimetric capacitance displayed an inverse relationship with the deposition time, as shown in Table 1 for the scan rate of 20 mVs^−1^, dropping from the initial 214 Fg^−1^ at 1 min deposition time to 126, 72, 27, 12, and 10 Fg^−1^ for the electrodes deposited for 2, 3, 7, 10, and 15 min, respectively. This decreasing capacitance with deposition time can be attributed to increased mass loading and thickness, restrict ionic diffusion for capacitive charge storage at greater depths within the electrodes. There is a direct relationship between deposited mass and an inverse relationship with specific capacitance (Fig. 8c). Notably, a decelerated kinetics for mass gain is observed after 10 min of electrodeposition, likely dpolymerue to the enhancement of the ohmic barrier by the growing layer between the substrate and the electrolyte [144,145]. The significant reduction in specific capacitance could be attributed to the formation of thicker electrode layers that may not significantly contribute to additional charge storage. Additionally, the argument of increased diffusion limitations as the cause of weakened behavior in more heavily loaded electrodes during capacitive performance is further supported by the observed trend in rate capability, which decreases from its maximum of 66 % for 0.1-M-1 to 49 % for 0.1-M-3 (see Table 1), indicating that the increase in deposition time and thickness leads to a higher fraction of non-superficial and non-contributing mass for capacitance [[146], [147], [148]]. This interpretation is consistent with the findings of Toupin et al. [149], who demonstrated that in thick MnO_2_ electrodes, only a thin surface layer is electrochemically active during the redox process, while the bulk remains largely inactive, contributing minimally to charge storage. Similarly, the work by Pang et al. [150] highlights the critical importance of optimizing film thickness, where thinner MnO_2_ films exhibit significantly better capacitive behavior due to enhanced ion accessibility and reduced diffusion limitations. These studies collectively support the idea that excessive deposition times, leading to thicker layers, can hinder electrochemical performance by increasing the proportion of inactive material and reducing both capacitance and rate capability.

**Fig. 9:**
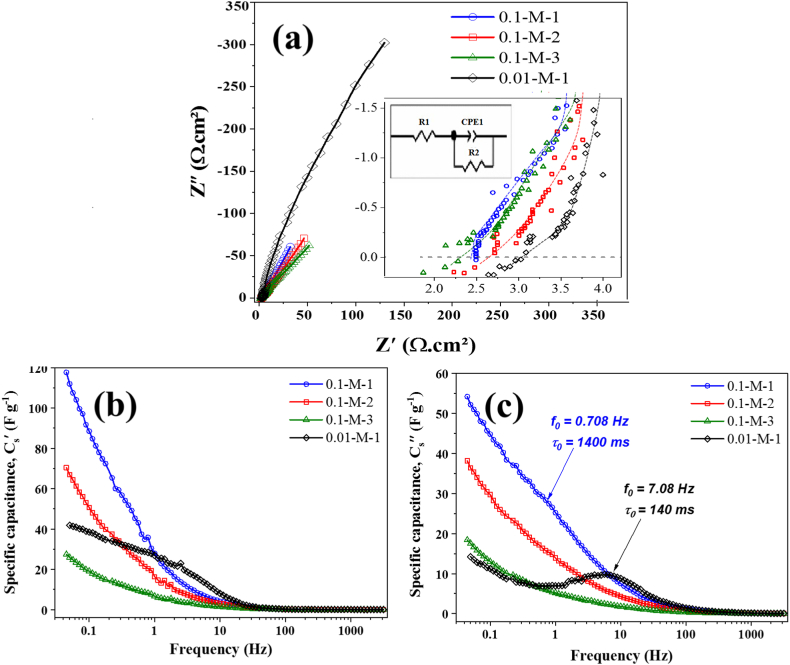
Nyquist plots of 0.1-M-x (1, 2, and 3), and 0.01-M-1 electrodes (inset: high-frequency region of the Nyquist plots and equivalent circuit of 0.01-M-1 electrode) (a), gravimetric real component of the complex capacitance, ${C_{s}}^{\prime }\left(f\right)$ (b), and gravimetric imaginary component of the complex capacitance, ${C_{s}}^{\prime \prime }\left(f\right)$ (c).

Fig. 6d–f shows the GCD curves of 0.1-M-x electrodes (x = 1, 2, and 3 min) at specific currents ranging from 1 to 5 Ag^–1^, revealing the rate sensitivity of each electrode in the mentioned window of charging rate. On the other hand, Fig. 6h compares the GCD behavior of different electrodes (for x = 1, 2, 3, 7, 10, and 15) at a fixed specific current of 1 Ag^–1^. Table 2 compares the specific capacitance of all electrodes (x = 1, 2, 3, 7, 10, and 15) at various specific currents. Additionally, Table 2 includes the ratio of the lowest specific capacitance (at the highest specific current, i.e., 5 Ag^–1^) to the highest one (at the lowest specific current, i.e., 1 Ag^–1^), serving as a measure of the electrode's capability to retain capacitance as the charging rate increases. A higher percentage ratio indicates a greater rate capability of the electrode. Overall, the GCD results are in complete agreement with the CV results, indicating that both the specific capacitance and the rate capability have an inverse relation with the deposition time.

**Table 2:** The specific capacitance and rate capability of the prepared samples based on the GCD characterization.

Electrode	C (Fg^−1^) at 1 Ag^–1^	C (Fg^−1^) at 2 Ag^–1^	C (Fg^−1^) at 3 Ag^–1^	C (Fg^−1^) at 5 Ag^–1^	C (Fg^−1^) at 10 Ag^–1^	Rate capability (%)
0.1-M-1	141	130	127	92	–	65
0.1-M-2	116	86	70	56	–	48
0.1-M-3	60	44	36	31	–	52
0.1-M-7	17	–	–	–	–	–
0.1-M-10	6	–	–	–	–	–
0.1-M-15	4	–	–	–	–	–
0.01-M-1	107	107	107	104	96	90

The impact of electrodeposition bath concentration on the capacitive behavior of the MnO_2_ coated layers was investigated by varying the concentration of KMnO_4_ aqueous electrolyte from 0.01 to 0.1 M. Fig. 7a and b presents the CV and GCD results of the electrode prepared with a 1 min electrodeposition time and 0.01 M KMnO_4_ bath (0.01-M-1 electrode). The CV scan rates range from 10 to 100 mVs^−1^, and GCD-specific currents range from 1 to 10 Ag^–1^. A qualitative comparison of the 0.01-M-1 electrode with the 0.1-M-x family suggests that the former electrode exhibits stronger characteristics of an intercalation-type electrode. Specifically, the peak/feature observed at the potential of ≈350 mV_Ag/AgCl_ in both CV and GCD plots, corresponding to the electron transfer in the Mn^3+^/Mn^4+^ redox system within the manganese oxide matrix triggered and promoted by the deinsertion/insertion of electrolyte cations into the oxide lattice pores (tunnels) [42,43,47,151,152], is prominent and intense in the 0.01-M-1 electrode. In contrast, it appears only as a weak feature in the 0.1-M-x electrodes. This observation lends support to the XRD and Raman interpretations, suggesting a δ-MnO_2_ layered structure with 2D tunnels for the 0.01-M-1 electrode, which facilitates ionic diffusion within the lattice pores.

The specific capacitance and rate capability values of the 0.01-M-1 electrode, obtained from CV and GCD tests, are presented in Table 1, Table 2, respectively. The specific capacitance of the 0.01-M-1 electrode, in the range of 80–90 Fg^–1^ calculated from CV and in the range of 95–105 Fg^–1^ calculated from GCD results, is generally lower than the optimal values observed for the 0.1-M-1 electrode. Likewise, are the energy and power densities of the 0.01-M-1 electrode, exhibiting lower values (3.45 Wh kg^−1^ and 228 W kg^−1^, respectively, at 1 Ag^–1^ and 0.5 V potential window) compared to 0.1-M-1 (4.51 Wh kg^−1^ and 184 W kg^−1^, at the same conditions). However, unlike the 0.1-M-1 electrode, the 0.01-M-1 electrode exhibits a significant rate capability across a wide range of experimental conditions, with the capacitance showing minimal dependence on the charging rate in both CV and GCD results (see Fig. 8a and b). In some instances, even slight enhancements are observed at higher rates, likely attributed to conditioning and pore-opening mechanisms [153]. Compared to the 0.1-M-x electrodes, which exhibit capacitance drops in the range of 35–50 % with increasing rates (Fig. 8a and b), the rate capability of the 0.01-M-1 electrode is remarkable. As indicated by the XRD and Raman results, the 0.01-M-1 electrode possesses a birnessite layered structure (δ-MnO_2_) with 2D tunnels separated by relatively large gaps, suitable for ionic diffusion and thus, reducing rate sensitivity and enhancing rate capability in supercapacitors [42,43,46,48]. Furthermore, Fig. 8c shows that the combination of low bath concentration and short electrodeposition time results in lower mass loading and thickness, thereby positively affecting the rate capability of the electrode [147].

The effects of deposition time and electrodeposition bath concentration on the capacitive performance of MnO_2_ have been previously discussed. However, examining capacitive behavior as a function of mass loading provides another valuable perspective. Increases in mass loading can result from either higher electrolyte concentrations or longer deposition times, but the underlying mechanisms and resulting electrochemical impacts differ between these scenarios. When mass loading increases due to higher electrolyte concentration, it is typically accompanied by increased density and reduced porosity, leading to a more compact morphology and crystalline structure with lower permeability. These characteristics negatively affect rate capability, as the denser structure impedes ion transport and limits active site accessibility at higher scan rates. However, the effect on capacitance is more nuanced. In low-rate tests, these dense structures can exhibit high capacitance (as shown in Table 1, Table 2, and Fig. 8, where the 0.1-M-1 electrode demonstrates higher capacitance but lower rate capability than the 0.01-M-1 electrode). This is likely because the denser coating provides a greater volume and surface area of micropores (both textural and lattice), which contribute to capacitance at lower rates, even though their effectiveness diminishes at higher scan rates. Differently, when mass loading increases due to prolonged deposition time, the primary effect is an increased MnO_2_ layer thickness, with minimal changes in morphology, density, porosity, or crystal structure. This results in a simultaneous reduction in capacitance and rate capability, driven by increased ion diffusion length, higher diffusion resistance, and decreased active material utilization as electrolyte penetration into the thickened electrode becomes more limited (as observed in the 0.1-M-3 electrode compared to the 0.1-M-1 electrode, Table 1, Table 2, and Fig. 8). Ragone plot of 0.1-M-x (x = 1, 2 and 3) and 0.01-M-1 indicates that the energy density of the sample has a constant and stable trend compared to other samples with increasing power density (Fig. 8d).

To provide a comparative evaluation of our work with other studies, particularly regarding rate capability, we conducted a comprehensive literature review on the pseudocapacitive behavior of MnO_2_-based supercapacitor electrodes. This review included electrodes fabricated via electrodeposition as well as other fabrication methods. Additionally, we considered studies on MnO_2_ composite electrodes and multi-phase architectures, which often demonstrate enhanced rate capabilities. The results of the review are summarized in Table 3. To ensure meaningful comparisons across studies with varying experimental conditions, we introduced several innovative metrics beyond the common "percentage capacitance retention or loss over a range of rates." For instance, the column detailing the "Capacitance drop (%) per order of magnitude rise in rate" offers a robust criterion for comparison. The capacitance drop (value or percentage), means the loss of capacity due to an increase in specific current or scan rate from lowest to highest rate (CV or GCD). As shown in the table, only a few studies (e.g. Refs. [42,68], and [154]) have achieved a rate capability comparable to ours, with a drop in capacitance of ≤10 % per order of magnitude increase in rate. This highlights the significance of our study in advancing the rate performance of MnO_2_ electrodes.

**Table 3:** Comparison of the rate capability figures of the 0.01-M-1 electrode in the present work with other literature reports on MnO_2_ supercapacitors.

MnO2 electrode description	Preparation method	MnO2 electrode class;	Electrochemical characterization method	R_L_, lowest rate (CV scan rate or GCD specific current)	R_H_, highest rate (CV scan rate or GCD specific current)	Orders of magnitude rise in rate	C_s,H_, highest specific capacitance	C_s,L_, lowest specific capacitance	Rate capability	Capacitance drop per order of magnitude rise in rate	Capacitance drop per order of magnitude rise in rate	Ref.
		Single-phase (SP), or Multi-phase hybrid/composite (MP)	CV, or GCD	mVs^−1^ or Ag^−1^ or A cm^−2^	mVs^−1^ or Ag^−1^ or A cm^−2^	Log_10_ (R_H_/R_L_)	F g^−1^	F g^−1^	%	F g^−1^	%	
MnO_2_ thin film on Ni foam	Electrodeposition	SP	CV	10	100	1.00	90	90	100 %	0	0 %	This work
MnO_2_ thin film on Ni foam	Electrodeposition	SP	GCD	1	10	1.00	107	96	90 %	11	10 %	This work
MnO_2_ nanoflowers on graphite	Electrodeposition	SP	GCD	1	40	1.60	314	192	61 %	76	24 %	[155]
MnO_2_ thin film on Ni nanorods	Electrodeposition	SP	CV	10	200	1.30	107	96	90 %	8	8 %	[68]
MnO_2_ coated on CNT	Electrodeposition pulsed current	MP	CV	5	1000	2.30	283	161	57 %	53	19 %	[37]
(Mn-Ni)O_2_ thin film on steel	Electrodeposition	SP	GCD	0.1	10	2.00	250	150	60 %	50	20 %	[144]
MnO_2_ thin film on steel	Electrodeposition	SP	GCD	0.1	10	2.00	235	115	49 %	60	26 %	[144]
MnO_2_ thin film on steel	Electrodeposition	SP	CV	5	100	1.30	200	150	75 %	38	19 %	[153]
MnO_2_ film, columnar structure, on Au-coated glass	Electrodeposition	SP	GCD	1	50	1.70	208	140	67 %	40	19 %	[89]
MnO_2_ film, interconnected sheets, on Au-coated glass	Electrodeposition	SP	GCD	1	50	1.70	229	171	75 %	34	15 %	[89]
MnO_2_ film, continuous coating, on Au-coated glass	Electrodeposition	SP	GCD	1	50	1.70	191	56	29 %	79	42 %	[89]
MnO_2_ nanorods, on carbon fiber paper	Electrodeposition	MP	GCD	0.5	5	1.00	363	160	44 %	203	56 %	[40]
MnO_2_ nanoflower, on carbon fiber paper	Electrodeposition	MP	GCD	0.5	5	1.00	236	100	42 %	136	58 %	[40]
MnO_2_ nanosheet, on carbon fiber paper	Electrodeposition	MP	GCD	0.5	5	1.00	226	85	38 %	141	62 %	[40]
MnO_2_ nanosphere, on carbon fiber paper	Electrodeposition	MP	GCD	0.5	5	1.00	134	75	56 %	59	44 %	[40]
MnO_2_ nanosheets on 3D-Ni network	Electrodeposition	MP	CV	5	125	1.40	370	250	68 %	86	23 %	[156]
MnO_2_ nanosheets on 3D-Ni network	Electrodeposition	MP	GCD	1	10	1.00	290	230	79 %	60	21 %	[156]
MnO_2_ film, nanoflakes, on Ni	Electrodeposition	SP	CV	1	50	1.70	170	85	50 %	50	29 %	[74]
MnO_2_ nanowhiskers on TiO_2_ Nts	Electrodeposition	MP	GCD	0.5	10	1.30	425	240	56 %	142	33 %	[82]
MnO_2_ nanowires on CNT paper	Electrodeposition	MP	GCD	0.075	0.75	1.00	170	110	65 %	60	35 %	[157]
MnO_2_ nanocrystals (antifluorite) on Pt	Electrodeposition	SP	CV	2	1000	2.70	190	100	53 %	33	18 %	[77]
MnO_2_ nanocrystals (rock salt) on Pt	Electrodeposition	SP	CV	2	1000	2.70	175	110	63 %	24	14 %	[77]
MnO_2_ nanocrystals (Ɛ) on Pt	Electrodeposition	SP	CV	2	1000	2.70	130	40	31 %	33	26 %	[77]
MnO_2_ nanosheet on CNT yarn	Electrodeposition	MP	GCD	0.001	0.015	1.18	3.6	2.1	58 %	1	35 %	[91]
MnO_2_ on graphene-CNT	Electrodeposition	MP	CV	10	400	1.60	330	265	80 %	41	12 %	[158]
MnO_2_-polypyrrole on carbon cloth	Electrodeposition	MP	CV	2	100	1.70	340	190	56 %	88	26 %	[159]
MnO_2_-polypyrrole on carbon cloth	Electrodeposition	MP	GCD	0.2	5	1.40	325	226	70 %	71	22 %	[159]
MnO_2_ on reduced graphene oxide	Electrodeposition	MP	GCD	1	20	1.30	347	175	50 %	132	38 %	[160]
MnO_2_ on Ni foam	Electrodeposition	SP	GCD	1	20	1.30	235	120	51 %	88	38 %	[160]
MnO_2_ nanotubes on Pt	Electrodeposition	SP	GCD	1	10	1.00	349	245	70 %	104	30 %	[161]
MnO_2_ on Ni foam	Electrodeposition	SP	GCD	0.002	0.02	1.00	200	125	63 %	75	38 %	[81]
(Mn-Cu)O_2_ on Ni foam	Electrodeposition	SP	CV	5	200	1.60	690	390	57 %	187	27 %	[162]
MnO_2_ nanowires on MnO_2_ nanofibers	Electrodeposition	SP	CV	50	1000	1.30	377	244	65 %	102	27 %	[93]
MnO_2_ film on FTO-Cu	Electrodeposition	MP	CV	5	50	1.00	486	350	72 %	136	28 %	[163]
MnO_2_ nano-sheets coated on MnO_2_ nano-spheres	Ultrasonic assisted chemical synthesis	SP	CV	5	100	1.30	309	107	35 %	155	50 %	[164]
MnO_2_ powder	Chemical	SP	GCD	0.5	10	1.30	297	257	87 %	31	10 %	[42]
MnO_2_ powder	Chemical	SP	CV	5	200	1.60	105	36	34 %	43	41 %	[55]
MnO_2_ powder	Hydrothermal	SP	CV	5	30	0.78	280	242	86 %	49	17 %	[57]
MnO_2_ powder	Hydrothermal	SP	GCD	0.2	1	0.70	571	140	25 %	617	100 %	[57]
MnO_2_ powder	Chemical	SP	GCD	0.5	2	0.60	170	147	86 %	38	22 %	[56]
MnO_2_-MgCo_2_O_4_	Multi-stage hydrothermal	MP	GCD	1	40	1.60	853	573	67 %	175	20 %	[38]
MnO_2_-Mn_2_O_3_	Multi-stage hydrothermal	MP	CV	1	100	2.00	240	85	35 %	78	32 %	[165]
MnO_2_-Mn_2_O_3_	Multi-stage hydrothermal	MP	GCD	0.05	5	2.00	192	96	50 %	48	25 %	[165]
MnO_2_-polypyrrole-carbon cloth	Multi-stage chemical hydrothermal	MP	CV	2.5	250	2.00	25	13	52 %	6	24 %	[36]
MnO_2_-polypyrrole-carbon cloth	Multi-stage chemical hydrothermal	MP	GCD	0.2	5	1.40	26	6.5	25 %	14	54 %	[36]
MnO_2_-graphene	Hydrothermal	MP	GCD	1	50	1.70	150	105	70 %	26	18 %	[166]
δ-MnO_2_ on β-MnO_2_	Chemical	SP	GCD	0.25	64	2.41	318	80	25 %	99	31 %	[167]
MnO_2_-graphene-CNT	Chemical	MP	CV	20	100	0.70	367	291	79 %	109	30 %	[168]
MnO_2_-CNT	Hydrothermal	MP	CV	50	800	1.20	62	45	73 %	14	23 %	[169]
MnO_2_-porous carbon spheres	Chemical	MP	CV	2	100	1.70	412	251	61 %	95	23 %	[170]
MnO_2_-graphene-CNT	Chemical	MP	CV	5	500	2.00	132	110	83 %	11	8 %	[154]

The promising rate capability of MnO_2_ electrodes prepared at low electrolyte concentrations and deposition times is primarily due to the significant impact of the electrolyte concentration on the microstructure, crystallinity, morphology, and porosity of the deposited layers. At lower electrolyte concentrations, the MnO_2_ films form with a highly porous and less dense structure, which facilitates rapid ion diffusion during electrochemical processes. Additionally, this condition favors the development of a layered δ-MnO_2_ phase with 2D tunnels that further enhance ionic transport. The morphology of the MnO_2_ also shifts towards a more open and interconnected columnar structure under low concentrations, which provides more accessible surface area for ion interaction. Furthermore, the short deposition time contributes to improved rate capability by producing thinner MnO_2_ layers. These thinner layers reduce the ionic diffusion length and resistance and ensure better utilization of the active material, allowing the electrode to maintain higher capacitance at increased scan rates. Together, these factors explain the enhanced performance of electrodes fabricated under these optimized conditions.

To further investigate the rate performance, frequency response, and electrical conductivity of the prepared electrodes, EIS measurements were carried out in the frequency range of 50 kHz to 50 mHz. The Nyquist plots for the y-M-x electrodes (y = 0.01 and 0.1 M; x = 1, 2, and 3 min) are shown in Fig. 9a. The equivalent series resistance (ESR, Ω) values were obtained from the intersection point of the Nyquist plot with the $Z^{\prime }$ axis in the high-frequency region, and then converted to areal resistance (Ω.cm^2^) by considering the approximate surface area of the Ni foams used for each electrode (≈800 m^2^ m^−3^, or ≈ 17 m^2^ g^−1^). Accordingly, areal ESR values of 2.5 ± 0.2, 2.6 ± 0.2, 2.4 ± 0.2, and 3 ± 0.2 Ω cm^2^, were calculated for 0.1-M-1, 2, 3, and 0.01-M-1 electrodes, respectively. The equivalent circuit of the 0.01-M-1 electrode is illustrated schematically in the inset of Fig. 9a.

A comparison between the 0.1-M-x electrodes reveals only minor variations (≈5 %) in ESR, which likely fall within the experiment's uncertainty margin. Consequently, the experimental resolution is insufficient for a rigorous evaluation of the effect of electrodeposition time and layer thickness on ESR. However, the 0.01-M-1 electrode shows an increase of about 0.5 Ω cm^2^ in ESR compared to the 0.1-M-x family, a difference larger than the experimental resolution. This rise likely stems from a deterioration in the electrical conductivity of the coating, assuming identical values for other factors contributing to the ESR, such as electrolyte conductivity and the distance between electrodes. The electrical properties of δ-MnO_2_ as reported in the literature [[171], [172], [173], [174]] are equivocal, with no consistent positive or negative conductivity behavior established as a general rule. Therefore, the observed ESR increase in 0.01-M-1 cannot be conclusively attributed to the structural transition from ε-MnO_2_ to δ-MnO_2_. Rather, it is more plausible that this increase results from the induced porosity and density loss in the electrode prepared at a lower electrodeposition bath concentration. Despite the slightly increased ESR of 0.01-M-1, and its presumably negative effect on rate performance, it is noteworthy that even the complex capacitance information (Fig. 9b and c), support evidence for the improved frequency response of this electrode. This finding aligns with the results from CV and GCD (Table 1, Table 2, and Fig. 8a–b), indicating that the ESR value remains within a reasonable range and does not significantly suppress the capacitive behavior.

Fig. 9b and c shows the gravimetric real and imaginary components of the complex capacitance, ${C_{s}}^{\prime }\left(f\right)$ and ${C_{s}}^{\prime \prime }\left(f\right)$, respectively. These were calculated from $C^{\prime }\left(\omega \right)$ and $C^{\prime \prime }\left(\omega \right)$ through simple mass normalization. The latter terms were derived from the EIS data using equations (5), (6)) respectively [175]:

(5) $$C^{\prime }\left(\omega \right)=\frac{-Z^{\prime \prime }\left(\omega \right)}{\omega {\left|Z\left(\omega \right)\right|}^{2}}$$

(6) $$C^{\prime \prime }\left(\omega \right)=\frac{Z^{\prime }\left(\omega \right)}{\omega {\left|Z\left(\omega \right)\right|}^{2}}$$

where, $C^{\prime }\left(\omega \right)$ and $C^{\prime \prime }\left(\omega \right)$ represent the real and imaginary components of the complex capacitance C(ω), respectively, $Z^{\prime }\left(\omega \right)$ and $Z^{\prime \prime }\left(\omega \right)$ are the real and imaginary components of the complex impedance Z(ω), respectively, |Z(ω)| is the magnitude (modulus) of impedance, and ω is the angular frequency (rad s^−1^), linked to the frequency, f (Hz), via ω=2πf.

While ${C_{s}}^{\prime }\left(f\right)$ represents the capacitive performance of the cell and reveals a similar trend in the capacitance ordering of the electrodes as those calculated from GCD or CV at intermediate–low frequencies (noting that the frequency window used here focuses more on faster phenomena, hence the numerical values of ${C_{s}}^{\prime }\left(f\right)$ at 50 mHz better match those obtained from GCD or CV at intermediate rates), ${C_{s}}^{\prime \prime }\left(f\right)$ is more useful for monitoring frequency-dependent energy dissipation in the system due to irreversible processes. In the ${C_{s}}^{\prime \prime }\left(f\right)$ plot, a peak appears at a characteristic frequency, known as the relaxation frequency ($f_{0}$), where the behavior of the supercapacitor shifts from predominantly resistive to capacitive. This peak is most evident for the 0.01-M-1 electrode and less clearly but still discernible for the 0.1-M-1, while the 0.1-M-2 and 0.1-M-3 electrodes lack a clear transition peak within the investigated frequency window, possibly due to the need for lower frequencies to enter the capacitive behavior dominance domain. The relaxation time $\tau_{0}$ (the reciprocal of $f_{0}$) of the first two supercapacitors is compared in Fig. 9c, shows that the time constant of the 0.01-M-1 electrode (≈140 ms) is approximately one order of magnitude shorter than that of the 0.1-M-1 (≈1.4 s). Notably, the observed frequency response of the 0.01-M-1 electrode is even faster than many carbon-based electrodes we have previously investigated (compare, for instance, with [148,176]) and is comparable with outstanding high-rate electrodes such as vertically aligned thin carbon nanotube films that were directly grown on metallic substrates [177]. Since $\tau_{0}$ reflects a balance between global resistance (encompassing all resistive elements such as electric and contact resistance of the active material, electrolyte resistance, ionic resistance in pores due to diffusion, charge transfer resistance of parasitic reactions, etc.) and global capacitance, it can be inferred that the advantages of the 0.01-M-1 electrode in terms of fast ionic transport outweigh the adverse effect of its slightly higher electrical resistance. Therefore, the superior frequency response of the 0.01-M-1 electrode is primarily attributed to the positive impact of its loose and open texture on ionic transport.

## 4 Conclusion

This study focuses on the electrodeposition technique as a simple and cost-effective method for preparing MnO_2_ coatings to serve as supercapacitor electrodes. To address the primary limitation of pseudocapacitive MnO_2_ electrodes, namely high rate sensitivity and low rate capability, aimed to optimize key electrodeposition parameters, including deposition bath concentration (ranging from 0.01 to 0.1 M KMnO_4_ aqueous electrolyte) and deposition time (ranging from 1 to 15 min).

The results demonstrate that reducing the electrodeposition bath concentration significantly enhances the rate performance of MnO_2_. At higher concentrations, hybrid oxide phases form, while lower concentrations favor the formation of birnessite δ-MnO_2_ with large interlayer spacing. Lower electrolyte concentrations also yield a more porous and less dense structure, featuring vertically aligned 2D columnar architecture that creates interconnected pores extending through the depth and thickness of the layer, thus enhancing ionic diffusion and surface accessibility for electrochemical reactions. Further analysis revealed that the apparent volumetric density of this structure (0.01-M-1 electrode, ≈1.25 g cm^−3^) is about one-quarter of the theoretical density of solid MnO_2_ (5.026 g cm^−3^) and roughly half that of the more concentrated electrolyte (0.1-M-1 electrode, ≈2.5 g cm^−3^).

The 0.01-M-1 electrode exhibited exceptional rate performance, maintaining a specific capacitance of 90–100 Fg^-1^ across various rates without significant degradation, as confirmed by Galvanostatic Charge-Discharge (GCD) tests (1–10 Ag^–1^) and Cyclic Voltammetry (CV) tests (10–100 mVs^−1^). This is a remarkable outcome, with our best-performing electrodes retaining ≥90 % of their capacitance per order of magnitude increase in rate –an accomplishment scarcely reported in the literature.

The superior rate performance of MnO_2_ electrodes at low concentrations and short deposition times can be attributed to several interrelated factors. The formation of a δ-MnO_2_ phase with a layered birnessite structure, characterized by large interlayer spacing (≥7 Å) and a highly porous morphology, facilitates rapid ionic transport at minimizing diffusion length and resistance, along with providing extensive surface area for electrochemical reactions. Thereby, it maintains a high capacitance at elevated scan rates.

In addition to structural and capacitive changes, electrodeposition parameters significantly impact MnO_2_ mass loading. Higher electrolyte concentrations increase mass loading through forming denser structures that enhance low-rate capacitance (e.g., 214 Fg^–1^ for 0.1-M-1 vs. 91 Fg^–1^ for 0.01-M-1 at 20 mVs^−1^) but reduce rate capability due to limited ion transport (e.g., 66 % vs. ≈100 % retention). Conversely, extended deposition times increase mass loading through increased layer thickness with minimal morphological and structural changes, leading to both lower capacitance and rate capability (e.g., 65 Fg^–1^ with 49 % retention for 0.1-M-3 vs. 0.1-M-1 electrode).

Looking ahead, several promising avenues for future research emerge from the insights gained in our study. These avenues aim to (1) maintain the excellent supercapacitive rate capability observed in the current work even at higher electrodeposition loadings/thicknesses, and (2) identify alternative MnO_2_ structures with potentially higher specific capacitance and equally strong rate capability. Firstly, exploring even lower electrolyte concentrations than those investigated here, though over longer durations, could further enhance our understanding of how reduced deposition kinetics impact the structural and electrochemical properties of MnO_2_ coatings. Additionally, employing deposition techniques with greater control over the deposition rate, such as galvanostatic or pulsed techniques, may offer alternative pathways to engineer the optimal microstructural architectures for improved rate capability. Furthermore, while our work focused on cathodic deposition to reduce the Mn oxidation state from +7 to +4, the opposite approach could also be explored –developing MnO_2_ coatings by increasing the Mn oxidation state from +2 in acetate or nitrate electrolytes, provided that kinetic feasibility assessments support the potential for achieving a desirably low deposition rate through anodic electrodeposition. Finally, incorporating additives to regulate deposition kinetics and optimize the structural characteristics of the films presents another valuable research direction. These approaches could lead to the development of advanced MnO_2_ electrodes with superior performance characteristics for supercapacitor applications.

## CRediT authorship contribution statement

**Hamed Soltani:** Writing – review & editing, Writing – original draft, Validation, Methodology, Investigation, Formal analysis, Data curation, Conceptualization. **Hamed Bahiraei:** Writing – review & editing, Validation, Supervision, Resources, Project administration, Investigation, Data curation, Conceptualization. **Shahnaz Ghasemi:** Writing – review & editing, Visualization, Validation, Supervision, Resources, Project administration, Methodology, Investigation, Formal analysis, Data curation, Conceptualization. **Mazdak Hashempour:** Writing – review & editing, Visualization, Validation, Formal analysis, Data curation, Conceptualization.

## Declaration of competing interest

The authors declare that they have no known competing financial interests or personal relationships that could have appeared to influence the work reported in this paper.
